# Seroprevalence of HIV, HBV, HCV and syphilis infections among blood donors at Gondar University Teaching Hospital, Northwest Ethiopia: declining trends over a period of five years

**DOI:** 10.1186/1471-2334-10-111

**Published:** 2010-05-10

**Authors:** Belay Tessema, Gizachew Yismaw, Afework Kassu, Anteneh Amsalu, Andargachew Mulu, Frank Emmrich, Ulrich Sack

**Affiliations:** 1 Department of Medical Laboratory Technology, College of Medicine and Health Sciences, University of Gondar, Gondar, Ethiopia; 2 Department of Microbiology and Parasitology, College of Medicine and Health Sciences, University of Gondar, Gondar, Ethiopia; 3 Division of Allergy and Clinical Immunology, Department of Medicine, University of Colorado Denver, USA; 4 Department of Medical Laboratory Technology, College of Medicine and Health Sciences, University of Hawassa, Hawassa, Ethiopia; 5 Institute of Clinical Immunology, Faculty of Medicine, University of Leipzig, Leipzig, Germany; 6 Fraunhofer Institute for Cell Therapy and Immunology, Leipzig, Germany; 7 Institute of Medical Microbiology and Epidemiology of Infectious diseases, Faculty of Medicine, University of Leipzig, Leipzig, Germany

## Abstract

**Background:**

Transfusion-transmissible infectious agents such as human immunodeficiency virus (HIV), hepatitis B virus (HBV), hepatitis C virus (HCV) and syphilis are among the greatest threats to blood safety for the recipient. This study aimed to determine the seroprevalence, risk factors and trends of HIV, HBV, HCV and syphilis infections among blood donors over a period of five years at Gondar University Teaching Hospital, Northwest Ethiopia.

**Methods:**

A retrospective analysis of consecutive blood donors' records covering the period between January 2003 and December 2007 was conducted. Logistic regression analysis was used to determine risk factors associated with HIV, HBV, HCV and syphilis infections.

**Results:**

From the total of 6361 consecutive blood donors, 607 (9.5%) had serological evidence of infection with at least one pathogen and 50 (0.8%) had multiple infections. The overall seroprevalence of HIV, HBV, HCV and syphilis was 3.8%, 4.7%, 0.7%, and 1.3% respectively. Among those with multiple infections, the most common combinations were HIV - syphilis 19 (38%) and HIV - HBV 17 (34%). The seropositivity of HIV was significantly increased among female blood donors, first time donors, housewives, merchants, soldiers, drivers and construction workers. Significantly increased HBV seropositivity was observed among farmers, first time donors and age groups of 26 - 35 and 36 - 45 years. Similarly, the seroprevalence of syphilis was significantly increased among daily labourers and construction workers. Statistically significant association was observed between syphilis and HIV infections, and HCV and HIV infections. Moreover, significantly declining trends of HIV, HCV and syphilis seropositivity were observed over the study period.

**Conclusions:**

A substantial percentage of the blood donors harbour HIV, HBV, HCV and syphilis infections. Strict selection of blood donors and comprehensive screening of donors' blood using standard methods are highly recommended to ensure the safety of blood for recipient.

## Background

The discovery of transfusion-transmissible infections (TTIs) has heralded a new era in blood transfusion practice worldwide with emphasis on two fundamental objectives, safety and protection of human life [1]. Blood safety remains an issue of major concern in transfusion medicine in Ethiopia where national blood transfusion services and policies, appropriate infrastructure, trained personnel and financial resources are inadequate.

Human immunodeficiency virus (HIV), hepatitis B virus (HBV) and hepatitis C virus (HCV) are of great concern because of their prolonged viraemia and carrier or latent state. They also cause fatal, chronic and life-threatening disorders. Blood transfusion accounts for 5-10% of HIV infections in sub-Saharan Africa [2]. Similarly, 12.5% of patients who received blood transfusion are at risk of posttransfusion hepatitis [3]. HBV is highly contagious and relatively easy to be transmitted from one infected individual to another by blood transfusion, during birth, by unprotected sex, and by sharing needles and has a relatively higher prevalence in the tropics [4,5]. A study conducted in Addis Ababa, Ethiopia showed that HCV antibody prevalence was 0.9% and higher among HIV-positive compared to HIV-negative individuals (4.5% vs. 0.8%, respectively). Similarly, higher prevalence of HCV antibodies was seen among HIV-positive compared to HIV negative antenatal care attenders (2.9% vs. 0.8%, respectively), and sex workers (5.3% vs. 1.3%, respectively) [6].

Syphilis is also a systemic disease caused by *Treponema pallidum *which can be spread by sexual contact, blood transfusion and via vertical transmission [7]. In sub-Saharan Africa, syphilis remains a serious public health problem. Prevalence of active syphilis infection among African countries showed 12.8% in Tanzania [8], and 3.8% in Kenya [9]. A study conducted to assess the prevalence of infection with HIV, syphilis and HBV among Ethiopian blood donors in 1995 showed that the seroprevalence of HIV-1, syphilis and HBV was 16.7%, 12.8% and 14.4%, respectively [10].

The high prevalence of HIV, HBV, HCV and syphilis has heightened the problems of blood safety in Ethiopia. Thus, continuous monitoring of the magnitude of transfusion-transmissible infections in blood donors is important for estimating the risk of transfusion and optimizing donor recruitment strategies to minimize infectious diseases transmission. Therefore, this study was conducted to determine the seroprevalence, risk factors and trends of HIV, HBV, HCV and syphilis infections among blood donors at Gondar University Teaching Hospital in Northwest Ethiopia.

## Methods

### Study design, setting and study subjects

A retrospective analysis of consecutive blood donors' records covering the period between January 2003 and December 2007 was conducted at Gondar University Teaching Hospital. The hospital is a tertiary level teaching hospital that provides health service to over five million inhabitants in Northwest Ethiopia, and is located 727 Km north from the capital city, Addis Ababa. Institutional ethical clearance was obtained from the research and publication committee of Gondar University. However, due to the nature of the study (retrospective review of blood donors' records), informed consent was not obtained from the study subjects.

Blood donors were either volunteers, or relatives or friends of patients and commercial donors who were recruited and paid by patients, their families, or friends to replace blood used or expected to be used for patients from the blood bank of the hospital. In the blood bank unit of the hospital, the first step in screening for potential blood donors is taking past medical history of the client. Individuals are required to give answers to a panel of questions on previous illnesses and medical conditions. Past history of blood transfusion and questions targeted to ascertain risky sexual behavior and practice are also part of the questionnaire. Apparently healthy subjects of age 17 to 65 years with body weight above 45 kg would qualify for donation. The medical and socio-demographic histories of the donors were recorded in the logbook and venous blood was collected in blood banking bags following standard procedures.

### Laboratory diagnosis for HIV 1 and 2

Each donor's serum sample was screened for HIV-1 and HIV-2 using Vironostika HIV Uni-Form II Ag/Ab (BioMerieux, Boxtel, The Netherlands) following the manufacturer's instructions.

### Laboratory tests for HBsAg and HCV antibodies

Sera were checked for the presence of hepatitis B surface antigen (HBsAg) using ELISA, Hepanostika HBsAg (Murex Biotech Ltd, Dartford, UK). Similarly, IgG antibodies to HCV were detected using an ELISA technique (Murex anti-HCV version 4.0) according to the manufacturer's instructions.

### Laboratory diagnosis for syphilis

Serum from all donors was tested for the presence of treponemal antibodies using rapid plasma reagin test (RPR) following the manufacturer's instructions (RPR, Wampole Laboratories, Princeton, N.J., USA).

### ABO blood grouping and Rhesus (RH) typing

ABO and Rh blood groups determinations were carried out on a slide using monoclonal blood grouping antisera; anti-A, anti-B, anti-AB, and anti-D (BIOTEC Laboratories Ltd, Great Britain).

### Statistical analysis

Data were entered, cleaned and analysed using SPSS version 13 statistical package. To ensure the quality of data entered into the computer, two people independently cross-checked each entry. Differences in prevalence of HIV, HBV, HCV and syphilis for Socio-demographic variables were tested for significance using logistic regression. Moreover, linear regression was used to assess the statistical significance of trends in seroprevalence of these pathogens over the study period. P value less than 0.05 was considered statistically significant.

## Results

### Demographic characteristics of donors

As shown in Table 1, a total of 6361 consecutive blood donors were screened at Gondar University Teaching Hospital blood bank unit during the study period. Of these, 5592 (87.9%) donors were males and 769 (12.1%) were females. The median age of the study subjects was 25 years (range 17 - 65 years). Of all donors, 3357 (52.8%) were in the age group of 17-25 years, 4372 (68.7%) were first time donors, 2952 (46.4%) were blood group O and 5984 (94.1%) were Rhesus D (RH) positive. In addition, daily labourers (27.8%), farmers (24.8%) and students, more frequently Collage/University students (21.2%) were constitute a major chunk of the blood donors. The relatively higher number of farmer and daily labourer donors may be due to the fact that farmers constitute a major portion of the general population and majority of the commercial blood donors are daily labourers.

**Table 1:** Socio—demographic characteristics of blood donors at Gondar University Teaching Hospital in Northwest Ethiopia 2003-2007

Characteristics	Number (%)
**Age group (years)**	
17 – 25	3357 (52.8)
26 – 35	1686 (26.5)
36 – 45	757 (11.9)
46 – 55	395 (6.2)
56 – 65	166 (2.6)
**Gender**	
Male	5592 (87.9)
Female	769 (12.1)
**Occupation**	
Daily labourer	1769 (27.8)
Farmer	1575 (24.8)
Student	1347 (21.2)
Housewife	212 (3.3)
Govt. employee	599 (9.4)
Merchant	333 (5.2)
Soldier	47 (0.7)
Driver	120 (1.9)
Construction worker	343 (5.4)
Others	16 (0.3)
**Number of donation**	
First donation	4372 (68.7)
Repeat donation	1989 (31.3)
**ABO Blood groups**	
O	2952 (46.4)
A	1715 (27.0)
B	1406 (22.1)
AB	288 (4.5)
**Rhesus (RH) type**	
Positive	5984 (94.1)
Negative	377 (5.9)

### Seroprevalence of HIV, HBV, HCV and syphilis

The overall seroprevalence rate of HIV, HBV, HCV and syphilis was 3.8%, 4.7%, 0.7% and 1.3% respectively (Table 2). Of all donated blood during the study period, 607 (9.5%) had serological evidence of infection with at least one pathogen and 50 (0.8%) had multiple infections. Among those with multiple infections, the most common combinations were HIV- syphilis 19 (38%) and HIV - HBV 17 (34%) (Table 3). As shown in Table 4, the seroprevalence of HIV was significantly increased among female blood donors (P < 0.001) compared to male blood donors, first time donors (P < 0.001) compared to repeat donors, and among housewives (P < 0.001), merchants (P < 0.001), soldiers (P = 0.020), drivers (P = 0.041) and construction workers (P = 0.009) compared to students. The seroprevalence of syphilis was significantly increased among daily labourers (P = 0.001) and construction workers (P = 0.013) compared to students. Similarly, the seropositivity of HBV was significantly increased among donors with the age groups of 26 - 35 and 36 - 45 years compared to the age group greater than 45 years, farmers (P = 0.005) compared to students and first time donors (P < 0.001) compared to repeat donors (Table 5).

**Table 2:** Trends of seropositivity of HIV, Syphilis, HBV and HCV among blood donors at Gondar University Teaching Hospital in Northwest Ethiopia 2003 - 2007

Year	Total screened N	HIV positive N (%)	Syphilis positive N (%)	HBV positive N (%)	HCV positive N (%)
2003	1156	58 (5.0)	45 (3.9)	62 (5.4)	X
2004	1693	61 (3.6)	33 (1.9)	85 (5.0)	23 (1.4)
2005	1187	47 (4.0)	1 (0.1)	63 (5.3)	7 (0.6)
2006	1045	33 (3.2)	2 (0.2)	30 (2.9)	3 (0.3)
2007	1280	40 (3.1)	2 (0.2)	58 (4.5)	2 (0.2)
Total	6361	239 (3.8)	83 (1.3)	298 (4.7)	35 (0.7)
p-Value of linear regression for trend	_	0.021	<0.001	0.061	<0.001

N = Number; X = Not done

**Table 3:** Prevalence of co-infections of HIV, HBV, HCV and syphilis among blood donors at Gondar University Teaching Hospital in Northwest Ethiopia 2003-2007

Co-infections	Number	Percent
HIV - Syphilis	19	38.0
HIV - HBV	17	34.0
HIV - HCV	4	8.0
HBV - Syphilis	7	14.0
HBV - HCV	2	4.0
Syphilis - HCV	1	2.0
**Total**	**50**	**100**

**Table 4:** Socio–demographic characteristics of blood donors by HIV and Syphilis sero positivity at Gondar University Teaching Hospital in Northwest Ethiopia 2003-2007

Characteristics	HIV Positive N (%)	OR (95% CI)	P-Values	Syphilis Positive N (%)	OR (95% CI)	P-Values
**Age group (years)**						
17 – 25	105/3357 (3.1)	1.03 (0.61 - 1.74)	0.902	31/3357 (0.9)	0.64 (0.30 - 1.41)	0.271
26 – 35	79/1686 (4.7)	1.57 (0.92 - 2.68)	0.096	28/1686 (1.7)	1.17 (0.53 - 2.58)	0.702
36 – 45	38/757 (5.0)	1.69 (0.94 - 3.03)	0.077	16/757 (2.1)	1.49 (0.63 - 3.51)	0.359
> 45	17/561 (3.0)	1.00	-	8/561 (1.4)	1.00	-
**Gender**						
Male	188/5592 (3.4)	1.00	-	76/5592 (1.4)	1.00	-
Female	51/769 (6.6)	2.04 (1.48 - 2.81)	<0.001	7/769 (0.9)	0.67 (0.31 - 1.45)	0.307
**Occupation**						
Student	34/1347 (2.5)	1.00	_	9/1347 (0.7)	1.00	_
Daily labourer	53/1769 (3.0)	1.19 (0.77 - 1.85)	0.429	38/1769 (2.1)	3.26 (1.57 - 6.77)	0.001
Farmer	56/1575 (3.6)	1.42 (0.92 - 2.19)	0.109	20/1575 (1.3)	1.91 (0.87- 4.21)	0.108
Housewife	26/212 (12.3)	5.40 (3.17 - 9.20)	<0.001	3/212 (1.4)	2.13 (0.57 - 7.95)	0.258
Govt.employee	17/599 (2.8)	1.13 (0.63 - 2.04)	0.689	0/599	-	-
Merchant	23/333 (6.9)	2.87 (1.66 - 4.93)	<0.001	4/333 (1.2)	1.81 (0.55-5.91)	0.327
Military	4/47 (8.5)	3.59 (1.22 -10.57)	0.020	0/47	-	-
Driver	7/120 (5.8)	2.39 (1.04 - 5.52)	0.041	1/120 (0.8)	1.25 (0.16 - 9.95)	0.833
Construction worker	19/359 (5.3)	2.16 (1.22 - 3.83)	0.009	8/359 (2.2)	3.39 (1.30 - 8.85)	0.013
**Number of donation**						
First donation	212/4372 (4.8)	3.70 (2.47 - 5.55)	<0.001	56/4372 (1.3)	0.94 (0.59 - 1.50)	0.803
Repeat donation	27/1989 (1.4)	1.00	-	27/1989 (1.4)	1.00	-
**HIV status**						
Positive	-	-	-	19/239 (7.9)	8.18 (4.81- 13.88)	<0.001
Negative	-	-	-	64/6122 (1.0)	1.00	-

OR = Odds ratio; CI = Confidence interval; N = Negative

**Table 5:** Socio–demographic characteristics of blood donors by hepatitis B and C virus seropositivity at Gondar University Teaching Hospital in Northwest Ethiopia 2003-2007

Characteristics	HBV Positive N (%)	OR (95% CI)	P-Values	HCV Positive N (%)	OR (95% CI)	P-Values
**Age group (years)**						
17 - 25	144/3357 (4.3)	1.43 (0.86 - 2.39)	0.166	16/2726 (0.6)	0.91 (0.26 - 3.12)	0.875
26 - 35	89/1686 (5.3)	1.78 (1.05 - 3.02)	0.032	13/1395 (0.9)	1.44 (0.41 - 5.08)	0.569
36 - 45	48/757 (6.3)	2.17 (1.23 - 3.81	0.007	3/621 (0.5)	0.74 (0.15 - 3.71)	0.718
> 45	17/561 (3.0)	1.00	-	3/463 (0.6)	1.00	-
**Gender**						
Male	273/5592 (4.9)	1.00	-	34/4528 (0.8)	1.00	-
Female	25/769 (3.3)	0.66 (0.43 - 0.99)	0.046	1/677 (0.1)	0.20 (0.03 - 1.43)	0.108
**Occupation**						
Student	55/1347 (4.1)	1.00	_	6/1188 (0.5)	1.00	_
Daily labourer	79/1769 (4.5)	1.10 (0.77 - 1.56)	0.602	8/1312 (0.6)	1.21 (0.42 - 3.49)	0.726
Farmer	102/1575 (6.5)	1.63 (1.16 - 2.28)	0.005	14/1293 (1.1)	2.16 (0.83 - 5.63)	0.117
Housewife	7/212 (3.3)	0.80 (0.36 - 1.79)	0.589	1/179 (0.6)	1.11 (0.13 - 9.25)	0.925
Govt.employee	20/599 (3.3)	0.81 (0.48 - 1.37)	0.432	2/525 (0.4)	0.75 (0.15 - 3.75)	0.729
Merchant	12/333 (3.6)	0.88 (0.47 - 1.66)	0.689	0/288	-	-
Military	2/47 (4.3)	1.04 (0.25 - 4.42)	0.953	0/34	-	-
Driver	4/120 (3.3)	0.81 (0.29 - 2.28)	0.689	0/103	-	-
Construction worker	17/359 (4.7)	1.17 (0.67 - 2.04)	0.585	4/283 (1.4)	2.82 (0.79 - 10.08)	0.110
**Number of donation**						
First donation	231/4372 (5.3)	1.60 (1.21 - 2.11)	<0.001	25/3610 (0.7)	1.11 (0.53 - 2.31)	0.79
Repeat donation	67/1989 (3.4)	1.00	-	10/1595 (0.6)	1.00	-
**HIV status**						
Positive	17/239 (7.1)	1.59 (0.96 - 2.64)	0.07	4/181 (2.2)	3.64 (1.27- 10.42)	0.02
Negative	281/6122 (4.6)	1.00	-	31/5024 (0.6)	1.00	-

OR = Odds ratio; CI = Confidence interval; N = Negative

The prevalence rate of syphilis, HBV and HCV among HIV infected donors was 7.9%, 7.1%, and 2.2% respectively, compared with the prevalence rate of 1.0%, 4.6% and 0.6% among HIV-seronegative donors. Furthermore, statistically significant association was observed between syphilis and HIV infection (P < 0.001) (Table 4), and HCV and HIV infection (P = 0.002) (Table 5).

### Trends of HIV, HBV, HCV and syphilis seroprevalence

Significantly declining trends of HIV (P = 0.021), HCV (P < 0.001) and syphilis (P < 0.001) seroprevalence were observed over the five years study period. The seroprevalence of HIV was 5.0% in 2003 and decreased to 3.6% in 2004 and slightly increased to 4.0% in 2005 but subsequently decreased to 3.2% in 2006 and 3.1% in 2007. The seroprevalence of HBV was decreased from 5.4% in 2003 to 2.9% in 2006 and increased further to 4.5% in 2007. HCV prevalence decreased steadily from 1.4% in 2004 to 0.6% in 2005, 0.3% in 2006, and 0.2% in 2007. Similarly, the prevalence of syphilis decreased progressively from 3.9% in 2003 to 1.9% in 2004, 0.1% in 2005 and 0.2% in 2006 and 2007 (Table 2).

## Discussion

In this study, significantly declining trends of HIV, HCV and syphilis seroprevalence were observed among blood donors over the study period. This finding is consistent with the observed declining trend of HIV seroprevalence in the general population in Ethiopia [11], declining trend of HIV prevalence among blood donors in Ethiopia [12], declining trend of HIV prevalence among pregnant women, and declining trend of syphilis infections among pregnant women in Addis Ababa [13]. The initial rise in HIV seroprevalence among the blood donors represents the peak of the epidemic when denial was prevalent and little attention was paid to the disease. The subsequent decline in HIV seroprevalence may be due to the effect of the prevention programs that have been instituted in recent years [14].

The overall seroprevalence of HIV (3.8%) in this study is similar to the 3.8% seroprevalence in Ghana [15]. However, it is lower when compared with the 5.5% in Maiduguri [16], 10.6% in Nigeria [17] and 16.7% in Ethiopia [10]. The significantly increased HIV seropositivity among female donors compared to male donors is in accordance with the previous report [18]. This significantly increased HIV seropositivity among female donors might be due to their increased vulnerability to HIV infection as a result of biological, social and economic disadvantages related to their gender [19].

In the present study, the seropositivity rate of HIV and HBV was significantly increased among first time donors compared to repeat donors. This is in agreement with the previous studies [20-22]. The significantly increased HIV and HBV seroprevalence among first time donors might be due to the fact that people who regularly donate blood usually have a profile of low-risk of HIV and HBV infection because they were selected many times [23]. The significantly increased HIV seroprevalence among housewife donors is also consistent with previous study [24] which shows that these women acquired the infection from their partners, who commonly have sexual relations with other women without their partner's knowledge. The partner, considering her relationship to be monogamous, does not use protective methods to avoid infection.

The seroprevalence of HBV (4.7%) is lower than the previous reports, 10.4% in Nigeria [25], 15.0% in Ghana [15] and 14.4% in Ethiopia [10]. The seroprevalence rate of HCV (0.7%) is in agreement with values ranging between 0 and 1.4% reported from USA and Europe [26,27] and 0.9% in Ethiopia [6]. However, it is lower than the 2.8% in Ghana [28] and the 2.9% in Port Harcourt [29]. Similarly, the seroprevalence of syphilis (1.3%) in this study is lower than the 3.6% in Maiduguri [23], 7.5% in Ghana [30], 12.8% in Ethiopia [31] and 12.7% in Tanzania [32] but is higher than the 0.1% reported in Port Harcourt [33]. The reason(s) for the relatively lower rate of seroprevalence of HBV, HCV and syphilis in this study compared with other studies cannot be discerned. The improvement in technology might make current screening reagents to be more specific and reliable; and could also be a pointer that there are geographical differences in prevalence.

Significantly increased in seroprevalence of HBV was observed in the age groups of 26 - 35 and 36 - 45 years compared to the age group of greater than 45 years. This is in concurrence with previous reports by Baba *et al*. [16] and Ejele *et al*. [33] in which higher prevalence was observed among youths. This observation is worrisome since the most productive and economically viable age group of the populations is worst hit. There is the need for renewed intensification of preventive programmes aimed at high risk behavioural change.

In this study, high prevalence rate of HIV, HBV, HCV and syphilis co-infections was revealed among blood donors. However, none of the donors showed the presence of three or four markers. The HIV - HBV co-infection rate of 17/50 (34%) and the 19/50 (38%) HIV - syphilis co-infection observed in this study are higher than the result in Port Harcourt[33] and lower than the 40% HIV/HBV co-infection rate reported by Lodenyo *et al*. [34]. This high rate of co-infection and the statistically significant relationship between HIV and syphilis, and HIV and HCV infections might be due to the fact that these pathogens share common modes of transmission and risk groups [6,16,25,35].

## Conclusion

A substantial percentage of the blood donors harbour transfusion-transmissible infections, 9.5% with at least one pathogen and 0.8% with multiple infections. Transmission of transfusion-transmissible infections during the serologically negative window period still pose a threat to blood safety in environments where there is a high rate of transfusion-transmissible infection. Therefore, strict selection of blood donors with the emphasis on getting voluntary donors and comprehensive screening of donors' blood for HIV, HBV, HCV and syphilis using standard methods are highly recommended to ensure the safety of blood for recipient. The prevalence of HIV, HBV, HCV, and syphilis co-infection needs to be studied on a larger scale for the better understanding of the impact on clinical outcome and treatment response.

## Competing interests

The authors declare that they have no competing interests.

## Authors' contributions

**BT **was the primary researcher, conceived the study, designed, participated in data collection, conducted data analysis, drafted and finalized the manuscript for publication. **GY**, **AA and AM **assisted in data collection and reviewed the initial and final drafts of the manuscript. **AK, FE **and **US **interpreted the results, and reviewed the initial and final drafts of the manuscript. All authors read and approved the final manuscript.

## Pre-publication history

The pre-publication history for this paper can be accessed here:

http://www.biomedcentral.com/1471-2334/10/111/prepub
